# Supplementary material for: An open-source framework for large-scale, flexible evaluation of biomedical text mining systems
Source: J Biomed Discov Collab. 2008 Jan 29;3:1. doi: 10.1186/1747-5333-3-1 (PMC2276192; doi:10.1186/1747-5333-3-1)
Supplement: Additional file 1 — Evaluation platform source code. The additional file contains the software evaluation framework discussed in this paper. The most current release can be downloaded from SourceForge.net [1]. See the accompanying README file for instructions on installation and use. Also included in the distribution is a collection of UIMA wrappers for some commonly used BioNLP tools and annotated corpora. [file 1747-5333-3-1-S1.zip › bionlp_uima_repository-1.0.1/docs/api/edu/uchsc/ccp/uima/cc/summary/comparison/ComparisonGroup.html]

ComparisonGroup (Center for Computational Pharmacology Util/UIMA API)


|  |  |  |
| --- | --- | --- |
| **Overview** \| **Package** \| **Class** \| **Tree** \| **Deprecated** \| **Index** \| **Help** | |  |
| **PREV CLASS**   NEXT CLASS | **FRAMES**    **NO FRAMES**     **All Classes** |  |
| SUMMARY: NESTED \| FIELD \| CONSTR \| METHOD | DETAIL: FIELD \| CONSTR \| METHOD |  |


---


## edu.uchsc.ccp.uima.cc.summary.comparison Class ComparisonGroup

```
java.lang.Object
  edu.uchsc.ccp.uima.cc.summary.comparison.ComparisonGroup
```

---

``` public class ComparisonGroup extends Object ```

---

**Constructor Summary**

`ComparisonGroup()`


| **Method Summary** | |
| --- | --- |
| `void` | `addAnnotationGroup(AnnotationGroupID group)` |
| `List<AnnotationGroupID>` | `getAnnotationGroupList()` |
| `String` | `getDescription()` |
| `boolean` | `isGoldStandard()` |
| `boolean` | `isValid()` |
| `void` | `print(PrintStream ps)` |
| `void` | `setAnnotationGroupList(List<AnnotationGroupID> annotationGroupMembersList)` |
| `void` | `setDescription(String description)` |
| `void` | `setGoldStandard(boolean isGoldStandard)` |

**Methods inherited from class java.lang.Object**

`clone, equals, finalize, getClass, hashCode, notify, notifyAll, toString, wait, wait, wait`

**Constructor Detail**

### ComparisonGroup

```
public ComparisonGroup()
```


**Method Detail**

### isValid

```
public boolean isValid()
```

---


### getAnnotationGroupList

```
public List<AnnotationGroupID> getAnnotationGroupList()
```

---


### setAnnotationGroupList

```
public void setAnnotationGroupList(List<AnnotationGroupID> annotationGroupMembersList)
```

---


### addAnnotationGroup

```
public void addAnnotationGroup(AnnotationGroupID group)
```

---


### getDescription

```
public String getDescription()
```

---


### setDescription

```
public void setDescription(String description)
```

---


### isGoldStandard

```
public boolean isGoldStandard()
```

---


### setGoldStandard

```
public void setGoldStandard(boolean isGoldStandard)
```

---


### print

```
public void print(PrintStream ps)
```


---


|  |  |  |
| --- | --- | --- |
| **Overview** \| **Package** \| **Class** \| **Tree** \| **Deprecated** \| **Index** \| **Help** | |  |
| **PREV CLASS**   NEXT CLASS | **FRAMES**    **NO FRAMES**     **All Classes** |  |
| SUMMARY: NESTED \| FIELD \| CONSTR \| METHOD | DETAIL: FIELD \| CONSTR \| METHOD |  |


---
